# Peer Intervention in Obesity and Physical Activity: Effectiveness and Implementation

**DOI:** 10.1007/s13679-025-00625-z

**Published:** 2025-04-03

**Authors:** Keith J. Topping

**Affiliations:** 1 Education and Society, University of Dundee, Dundee, Scotland DD1 4HN UK; 2 Birch Tree Lodge, Meadowbeck Close, York YO10 3SJ UK

**Keywords:** Peer intervention, Peer education, Peer counseling, Peer support, Developing countries, Implementation

## Abstract

**Purpose of Review:**

This paper reports the effectiveness of peer intervention in physical activity and obesity, with a focus on implementation. Peer intervention is a parallel method to traditional professional clinical processes, often targeting hard to reach populations. It includes peer education, peer counseling and peer support.

**Recent Findings:**

There were ten reviews on Physical Activity and seven on Obesity. Six reviews on obesity had mainly positive results; one on obesity in mental health was more negative. About two-thirds of reviews of Physical Activity interventions had positive outcomes. The overall effect was moderate. There were 39 single studies on Obesity and 46 on Physical Activity. 36% of Obesity studies and 13% of Physical Activity studies were from developing countries. Three single studies from developing countries and three from developed countries were elaborated. The extensively described implementation program was from a developed country.

**Summary:**

Discussion of limitations and strengths led to recommendations for implementation and evaluation. Overall, peer intervention in both obesity and physical activity showed quite strong evidence of effectiveness. Had all studies followed the implementation/evaluation recommendations, the strength of evidence might have been better. Future research should focus on cost-effectiveness and long-term follow-up.

**Graphical Abstract:**

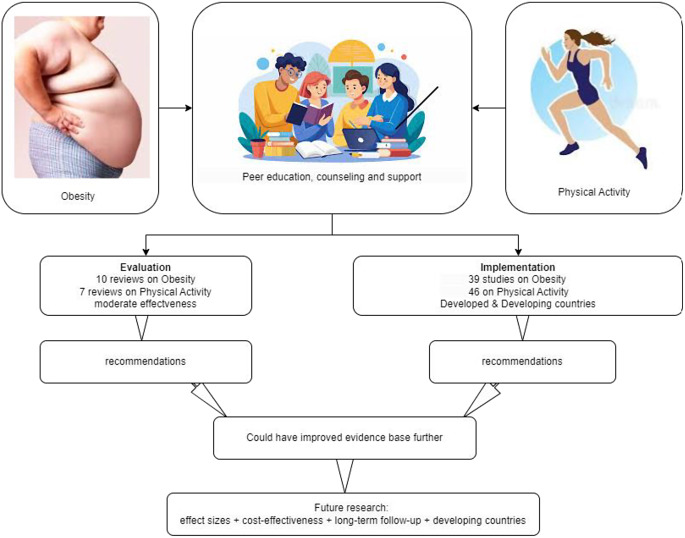

**Supplementary Information:**

The online version contains supplementary material available at 10.1007/s13679-025-00625-z.

## Practitioner Points

1.  Peer intervention includes peer education, peer counseling and peer support.
2.  Peer intervention in both obesity and physical activity showed quite strong evidence of effectiveness (moderate effect sizes).
3.  Had all studies followed the implementation/evaluation recommendations, the strength of evidence might have been better.

**Supplementary Information:**

The online version contains supplementary material available at 10.1007/s13679-025-00625-z.

## Introduction

As global diets have changed in recent decades, there has been an increase in the consumption of foods high in fat and sugar, leading to obesity in some people. There has also been a decrease in physical activity due to the changing nature of many types of work and easier access to transportation. Worldwide adult obesity has more than doubled since 1990: in 2022 2.5 billion adults were overweight and 890 million were living with obesity (one in eight people in the world). Since 1990, adolescent obesity has quadrupled: in 2022, over 390 million children and adolescents aged 5–19 years were overweight, including 160 million living with obesity [1]. Obesity is a risk factor for heart disease and stroke, diabetes and osteoarthritis, so interventions to increase physical activity have become more widespread, to prevent or reduce obesity.

## Aim

This paper aims to report the effectiveness of evidence-based methods and programs in peer intervention for health and well-being in obesity and physical activity. Although it includes a review of reviews, it is not a traditional systematic review or meta-analysis, and has a further strong focus on implementation. Peer intervention is an alternative or parallel method to traditional professional clinical processes, which often targets hard to reach populations. After a definition of terms, the paper offers a review of reviews of the evidence on the effectiveness in peer intervention in obesity and physical activity, then moving on to discuss key single papers, then describing implementation procedures from one study in detail, to help practitioners who are likely to ask “yes, but how do we actually do it?”. This paper stems from a recent book [2] which includes chapters on practical implementation and evaluation methods. It is coupled with an extensive list of individual papers, available only online.

## Context

Arguably, peer intervention has more chance of permeating the peer group and changing behavior than simple information-giving by professionals. Peer intervention can bridge the gap between professional clinical advice and the “real world”, reaching where professionals cannot be or cannot go. Many programs find gains for peer helpers as well as the helped; to be a helper may actually be more therapeutic than being helped. Of course, peer intervention is not always a stand-alone program– often it complements more traditional professional services. Many medical personnel will have had training and experience that focuses on what they can do directly to help patients. This paper takes a different angle - what can medical personnel (and others) do to train and support peers to work effectively with patients to support and guide them in a more informal way, often in language they are more likely to understand, and often based on the helper’s own experience of problems closely related to those the targeted person is suffering?

## Definitions

Peer intervention principally includes peer education, peer counseling and peer support (although other labels such as peer mentoring, peer coaching or peer navigation are sometimes used). Peer education can be defined as peers offering credible and reliable information about sensitive life issues which impact health and well-being and the opportunity to discuss them in a one-to-one or informal peer group setting. Peer counseling can be defined as people from similar groupings who are not professionals who help to clarify life problems and identify solutions by listening, clarifying, feeding back, summarizing, questioning and being positive, supportive and reassuring - and then helping plan, organize and problem-solve. Peer support is more difficult to define. It often has helpers use their own experiences to help the recipient. It is intended to introduce the patient to ideas and approaches that others have found helpful and reassure the patient that they are not alone in how they are feeling. It aims to provide a space where patients feel accepted and understood. Peer counseling and support might involve meeting in person or be accessed online– for example through social media networks or communities, emails, phone calls or text messages [2].

In all of these, “peer” means someone of approximately similar background and age who has no professional training in the area in question, but may well have experienced the issues being discussed at first hand. Peer interventionists should have benefited from training to ensure the facts they are transmitting are accurate. Peer education is usually in groups, while peer counseling may be one-to-one, in groups or a mixture of the two. Peer support is usually one-to-one. However, many authors make no attempt to define activities as one or the other. As the terms peer education, counseling and support are used very vaguely in the literature and are often interchangeable, no effort is made here to analyze them separately.

## Overview of Effectiveness of Peer Intervention

Although peer intervention has been used widely for over fifty years, for many of these years the background research was weak. However, now the background research features many strong overall reviews in a number of areas, particularly in more recent years. Of these, a few were meta-analyses and the rest equally divided between narrative reviews and systematic reviews. About a third focused on young people, another third on adults and the remainder on a mixture of the two. About two-thirds were about preventive interventions while the remainder were about corrective interventions. Later reviews were more likely to include Randomized Controlled Trials (RCTs). Several reviews found peer intervention to be as effective as or more effective than traditional professional clinical advice, at least in the populations under investigation, although the majority did not make such a comparison [2]. However, there were many other programs and methods which had not been evaluated at all and these approaches are not mentioned further.

## Search Methods

**Fig. 1:**
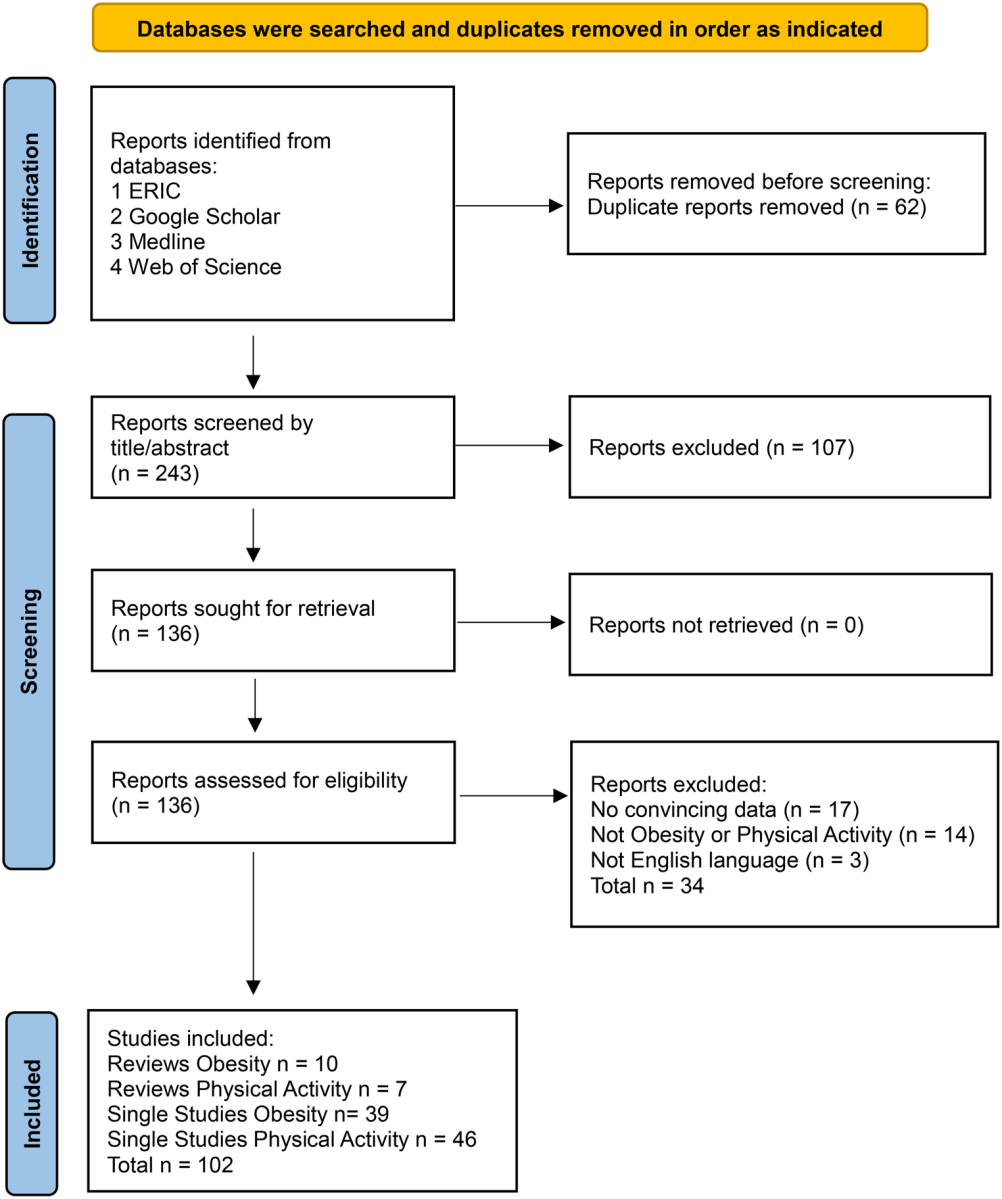
PRISMA Flowchart for Obesity and Physical Activity

Four relevant research databases (ERIC, Google Scholar, Medline and Web of Science in that order) were searched for peer-reviewed journal papers using the terms: “peer education” OR “peer counseling” OR “peer counseling” OR “peer support” AND Obesity OR “Physical Activity”. Hits were extracted up to the point where ten consecutive pages in each database had no relevant hits. Campos, et al. [3] found that 95% of all relevant abstracts within a given dataset could be retrieved using heuristic rules such as stopping the screening process after classifying 20% of records. The fewest hits were found in ERIC, most hits were found in Google Scholar and Medline, and given the order, Web of Science struggled to generate new hits not otherwise found (see Fig. 1). Papers were investigated which met the inclusion criteria: written in the English language; peer reviewed journal article; dated 2000 to present day; focusing on peer intervention to promote health or well-being; being a systematic analysis or meta-analysis or including quantitative and/or qualitative data to support the conclusions regarding effectiveness. The full text was read and the paper selected or de-selected. Papers were categorized into those reporting on developed countries and those reporting on developing countries, since the contexts are often very different. Papers from developing countries were sometimes not as scientifically rigorous as papers from developed countries.

## Reviews of Evidence on Obesity and Physical Activity

There were seven reviews on Obesity and ten reviews on Physical Activity. It is interesting that there were more reviews on preventive than corrective concerns. The majority of studies on Physical Activity came from developed countries, which was not true of Obesity.

### Obesity

Nickols-Richardson et al. [4] conducted a systematic review from 1977 to 2013 on nutrition‐ and obesity‐related outcomes in youth. They included 15 studies, reporting various methods of selecting, training and assigning responsibilities to peer educators. There were some studies.

showing peer education improved attitudes, perceptions and self-efficacy for healthy eating, but nothing using physiological indicators. A narrative review of results from youth peer-led lifestyle modification and weight management programs was offered by Vangeepuram et al. [5]. However, they only searched one database from 2002 to 2015, identifying 29 interventions for children from kindergarten to 12th grade. Youth peer-led interventions could result in positive changes in behavior, diet, physical activity, body measures and other clinical outcomes, but a minority of studies showed no positive results. It was recommended that peer leaders receive extensive training and regular supervision.

Studies then began to include physiological indicators. Lim et al. [6] aimed to meta-analyze the efficacy of peer interventions on body weight, energy intake and physical activity in adults, searching 14 databases and including 65 articles. Overall, peer interventions resulted in significant reduction in weight (28 studies), BMI (25 studies), waist circumference (12 studies) and a significant increase in physical activity (41 studies), but with no significant effect on energy intake. Mean differences and probabilities were cited but not standardized mean differences or effect sizes. In the same year Chen et al. [7] conducted a meta-analysis on weight, BMI, waist circumference, blood pressure, quality of life, social support and depressive symptoms. They searched three databases to 2020 and included 14 RCTS. A significant improvement in weight was found in individuals who received peer support compared to usual care (*p* = 0.02). Peer support was also associated with significant decrease in BMI (*p* = 0.04). Again, mean differences and probabilities were cited but not standardized mean differences or effect sizes. However, there was no statistically significant improvement in the levels of waist circumference, systolic blood pressure, diastolic blood pressure, quality of life, social support and depressive symptoms.

Martin-Vicario et al. [8] reviewed two databases from 2011 to 2021, selecting 21 studies and focusing on the effects of social support for members of obesity online health communities.

Twelve of 21 studies showed a positive relationship between social support and weight loss, the reminder not dealing directly with weight loss but no study showing negative results. Social support also improved behavioral change and community participation. This study pointed to both a direct and indirect relationship between social support in online health communities and actual weight loss. A meta-analysis of peer-led interventions on child and adolescent obesity was reviewed by Nguyen et al. [9]. They included 14 studies with a minimum duration of four weeks and reporting on BMI, all from high-income countries. The meta-analysis showed effectiveness (*p* = 0.01). There were significantly greater effects from interventions focusing on physical activity alone or with longer duration of implementation. Again, mean differences and probabilities were cited but not standardized mean differences or effect sizes.

One outlier review focused on obesity in people with serious mental illness (Stubbs et al. [10]), investigating peer intervention effects on physical health, lifestyle factors and appointment attendance. A systematic search to 2016 resulted in seven studies being included, including three RCTs. There was considerable heterogeneity in the type of peer support intervention and the role of the peer support workers. Three studies found insignificant reductions in weight. Evidence from another three studies on lifestyle changes was equivocal, only one study demonstrating improved self-reported physical activity and diet. Evidence regarding appointment attendance was also unclear across four studies. No effect sizes were reported.

### Physical Activity

Ginis et al. [11] included ten published studies. In all studies reporting within-group analyses, peer-delivered interventions led to increases in physical activity. Peer-delivered interventions were just as effective as professionally delivered interventions and more effective than control conditions for increasing physical activity. Three studies yielded evidence that peers may also enhance self-efficacy and self-determination. One study showed sustained gains at 18-month follow-up. No effect sizes were cited. School-based peer interventions were systematically reviewed by Jenkinson et al. [12]. Only four RCTs were identified and only two other studies had control groups. Nine of the 19 studies reported significant findings. Tutor training varied considerably; 13 of the 19 studies provided some training. No effect sizes were given. Mendonca et al. [13] searched eight databases to 2011 and 75 studies were included, mainly using self-report methodologies. Social support was consistently associated with the physical activity level of adolescents in cross-sectional and longitudinal studies, but the direction of causality was not clear and no effect sizes were cited. Those who received more support showed higher levels of physical activity.

Three information databases in Iran were searched by Abdi et al. [14] from 2003 to 2013; 20 studies were included. Of twenty trials, seven were conducted in school/university and three in the workplace, while the others were conducted in clinical settings. All studies used self-reporting instruments. Only one study failed to find significant results, but only three were considered of high quality. No effect sizes were given but five studies addressed maintenance over different periods of time. Best et al. [15] systematically reviewed and meta-analyzed outcomes, searching six databases for RCTs and including 21 studies. Statistically significant improvements in physical activity were reported by 14 studies. A meta-analysis of 17 studies showed a statistically significant pooled effect size (0.4, *p* < 0.001) immediately post-intervention. A large effect was evident in the four studies that included follow-up measures (1.5, *p* = 0.03).

A focus on older people came from Burton et al. [16]. The systematic review and meta-analysis covered 1976–2016 from six databases including 18 studies. Average participant age was 66.5 years and 67% were female. Interventions included resistance, flexibility and cardiovascular training. Sixteen of the 18 studies reported improvement in levels of physical activity and/or noted physical benefits by peer involvement. Six studies suggested their peer led interventions may be as effective as those run by health professionals. No effect sizes were cited. Hulteen et al. [17] searched eight databases for five years from 2018, including 15 studies. Children and adolescents represented the target population in almost half of the studies (*n* = 7), with four of those studies conducted with only adolescent girls. In addition,

medical populations were targeted in five studies (breast cancer survivors, older adults with type 2 diabetes, veterans with mental illness, older adults with different chronic illnesses, and patients with hypertension). Eighty per cent of the studies reported a significant intervention effect on at least one measure of physical activity. Peer-delivered interventions appeared to represent an efficacious means of promoting physical activity among diverse populations across the lifespan and in different settings. No effect sizes were cited.

A scoping review by Tweed et al. [18] searched four databases 2007–2019, including 13 studies. Nine studies found a significant increase in perceived social support, seven studies reported increased mental wellbeing and five studies reported increased physical activity levels. Gains also included: exercise-related psychosocial benefits, knowledge relating to selfcare and improved social connections. Five studies reported gains were maintained at follow-ups which ranged from three to nine months. No effect sizes were given.

Christensen et al. [19] focused on youth, conducting a scoping review including 43 studies. Youth peer leadership initiatives could increase physical activity for youth and children.

Three studies reported significant intervention effects on physical activity mean counts per minute, average daily minutes of moderate to vigorous intensity and step counts per minute. Four studies reported intervention effects on fitness and object control skills measured with physical tests. Two studies reported a significant intervention effect on.

fitness and object control skills. Other studies tended to depend on self-report. No effect sizes were given. Similarly, McHale et al. [20] focused on adolescents, searching three databases for studies reporting at least one outcome in 12- to 19-year-olds and including 18 studies. Half (*n* = 9) reported improved outcomes in the school setting. Five interventions reported statistically significant increases in moderate-to-vigorous-physical-activity (MVPA). No effect sizes were cited. The most prominent behavioral change techniques were social support, information about health consequences and demonstration of the behavior. Both cross-age and same-age interventions seemed effective. Gender-specific interventions showed great promise.

### Single Studies

#### Number of Single Studies

There were 39 single studies on Obesity and 46 on Physical Activity. In Obesity, 14 out of 39 studies (36%) were from developing countries or focused on disadvantaged communities, (this relatively high proportion reflecting the global concern with this issue), while 25 were from developed countries. In Physical Activity, six out of 46 studies (13%) were from developing countries or focused on disadvantaged communities, (this relatively low proportion reflecting a lesser preoccupation with preventive studies in developing countries), while 40 were from developed countries. The exemplar single studies were chosen from each context (developed/developing) in both obesity and physical activity as best illustrating differences in implementation. A full listing of the remaining single studies in provided in Supplementary Materials online.

#### Exemplar Single Studies Developed Context

Mothers in Motion (MIM), a community-based intervention program, was designed by Chang et al. [21] to help young, low-income women with overweight or obesity prevent further weight gain by promoting stress management, healthy eating, and physical activity. This study reports MIM’s effect on self-efficacy to cope with stress, emotional coping response, social support for stress management, stress, depressive symptoms, and positive and negative affect. Participants (*n* = 612) were recruited in Michigan USA and randomly assigned to an intervention group (410 participants) or comparison group (202). During the 16-week intervention, intervention participants watched ten video lessons at home and joined ten peer support group teleconferences. Surveys with established validity and reliability were used to measure self-efficacy to cope with stress, emotional coping response, and social support for stress management at the end of the 16-week intervention and at three-month follow-up. At post-test, the intervention group reported significantly higher self-efficacy to cope with stress (effect size d = 0.53), better emotional coping response (d = 0.38), less stress (d = 0.34), fewer depressive symptoms (d = − 0.27) and more positive affect (d = 0.31) than the comparison group. However, there were no significant differences in social support for stress management or negative affect. At follow-up, the intervention group still reported significantly higher self-efficacy to cope with stress (d = 0.32) and better emotional coping response (d = 0.34) than the comparison group, but did not report significantly higher social support for stress management, stress, depressive symptoms, or positive and negative affect.

Huitink et al. [22] noted that supermarkets located near schools influenced adolescents’ food consumption. Their study aimed to: (1) measure dietary behaviors during school hours, (2) to investigate the effect of a nutrition peer-education intervention in supermarkets within walking distance to secondary schools on nutritional knowledge and attitudes toward healthy eating, and (3) to assess how the intervention was appraised by adolescents with a lower education level. The participants were adolescents aged 12–14 years from four secondary schools (*n* = 432). Cross-sectional analyses were performed to establish dietary behaviors (pretest). A quasi-experimental pre–post design with a comparison school was used. Intervention schools received the intervention in a supermarket near their school. Most of the adolescents who purchased foods from retail food outlets near the school (71%) did so from supermarkets (89%). The nutritional knowledge scores (*p* = 0.003) as well as the attitudes toward healthy eating (*p* = 0.009) of adolescents from the intervention schools were statistically significantly higher after the intervention, relative to the comparison school.

Whether tailored support from older peer volunteers could improve initiation and long-term maintenance of physical activity behavior for older adults was examined by Burman et al. [23]. Participants were randomized to two 16-week, group-based programs: (1) peer-delivered, theory-based support for physical activity behavior change; or (2) an intervention typically available in community settings (basic education, gym membership, and pedometer for self-monitoring), attention-matched with health education. Moderate-to-vigorous physical activity (MVPA) was assessed via daily self-report logs at baseline, at the end of the intervention (16 weeks), and at follow-up (18 months), with accelerometry validation in a random subsample. Seven peer volunteers and 81 sedentary adults were recruited. Retention at the end of the trial was 85% and at follow-up at 18 months 61%. At 16 weeks, both groups had similar significant improvements in MVPA. At 18 months, the group supplemented with peer support had significantly more MVPA.

#### Exemplar Single Studies Developing Context

Jeihooni et al. [24] aimed to determine the effect of peer education on nutrition and physical activity in 200 female high school students who had obesity and overweight issues, randomly assigned to control and experimental groups. Peer intervention occurred in ten educational sessions of 50–55 min. The physical activity and nutrition performances questionnaire was completed before and three months after. Before educational intervention there were no significant differences between experimental and control groups in knowledge, attitude, self-efficacy, enabling factors, reinforcing factors, physical and nutrition performances. However, three months after the intervention, the experimental group showed significant enhancement in all of these compared to the control group. Pre-intervention there were no significant differences in weight and BMI between the experimental and control groups, but post-intervention there were significant differences in the experimental group, while control group had no changes.

Gunawardena et al. [25] developed a program that enabled school children to act as change agents in promoting healthy lifestyles in their mothers, measured by weight, physical activity and dietary habit. A 12-month cluster randomized trial was conducted, with school as a cluster. Participants were mothers of grade 8 students (aged around 13 years) in 20 schools in Sri Lanka. Students were trained to acquire the ability to assess non-communicable disease risk factors in their homes and take action to address them. Body weight, step count and lifestyle of the mothers were assessed at baseline and post-intervention. Of 308 study participants, 261 completed the final assessment at 12 months. There was a significantly greater decrease of weight and increase of physical activity in the intervention group. The intervention group had 3.25 times higher odds of engaging in adequate physical activity than the control group, and showed a greater number of steps than the latter. The effect size was 2.49 for weight and 0.99 for BMI. The intervention group showed a greater reduction of household purchase of biscuits and ice cream.

Older people with type 2 diabetes were studied by Sazlina et al. [26], who compared personalized feedback alone or with peer support. A three-arm randomized controlled trial was conducted in Malaysia. Sixty-nine sedentary Malays aged 60 years and older who received usual care were randomized to feedback or feedback + support interventions or served as controls for 12 weeks, with follow-ups at weeks 24 and 36. Intervention groups performed unsupervised walking activity and received written feedback on physical activity. The feedback + peer support group also received group and telephone contacts from trained peer mentors. The primary outcome was pedometer steps. Secondary outcomes were self-reported physical activity, cardiovascular risk factors, cardiorespiratory fitness, balance, quality of life and psychosocial wellbeing. Fifty-two participants (75%) completed the study. The feedback + peer support group showed greater daily pedometer readings than the feedback and control groups (*p* = 0.001). This group also had greater improvement in weekly duration (*p* < 0.001) and frequency (*p* < 0.001) of moderate intensity physical activity, scores on the Physical Activity Scale for Elderly (*p* = 0.003), six-minute walk test (*p* < 0.001) and social support from friends (*p* = 0.032).

### Program Illustrating Implementation in Greater Detail

The program illustrating implementation in greater detail was in obesity and other areas and was drawn from a developed country.

#### Effectiveness

A cluster RCT was conducted by Diao et al. [27], involving 1,564 subjects who were divided into an intervention group (*n* = 714) and a control group (*n* = 850). The intervention group received a full year of peer education. Their Quality of Life (QoL) was assessed using the Adolescent Quality of Life Scale and a self-designed basic situation questionnaire. After the intervention, significant increases were found in the psychological (*p* = 0.013) and in total QoL (*p* = 0.016) in the intervention group relative to the control group. However, no significant changes were observed for the social QoL of both groups and the total Qol of the control group (*p* > 0.05).

#### Implementation

The cluster RCT involved four schools (two primary schools, two junior middle schools) and were randomly divided into intervention and control groups. The study began in grades 4–5 (primary) and grades 7–8 (junior middle). The average age of the participants was 12.5 years. A total of 1,789 students were eligible to participate, but nine were excluded on the grounds of mental retardation. After one year of peer education a follow-up was carried out on the same subjects. The baseline survey was done with 787 intervention students and 993 control students, who completed a 40-minute QoL questionnaire. The reliability and validity of the QoL scale was assessed in other primary and junior middle schools, resulting in alpha coefficients of the physical, social, psychological and pubertal dimensions and whole scale at 0.81, 0.77, 0.85, 0.64 and 0.89, respectively. The retest reliability values were 0.76, 0.78, 0.82, 0.72 and 0.88, respectively. The intervention was then initiated and completed three months later. The final assessment was conducted nine months later.

Following discussions with class teachers, the researchers selected four excellent, prestigious and well-communicated students (two boys and two girls) who were class committee members and actively participated in extracurricular activities, who were asked to act as peer educators. Adolescent health education training for the peer educators was conducted and its effectiveness evaluated. The results showed the correct knowledge and the attitudes of the peer educators significantly improved. Secondly, the forms of peer education included knowledge contests, group discussions, role-playing and self-made poster exhibitions based on adolescent health education (knowledge quiz software was provided, as were group discussion cases and analysis results, role-playing scripts, and related pubertal health education slides). Peer educators utilized the class meeting or spare time to perform related activities amongst their peers. After each activity, the peer educators recorded key information including the number of participants, the content of the discussions and whether the peers showed relevant knowledge acquisition. Each class was required to have at least two activities per month. Thirdly, secondary peer educator training was performed to consolidate relevant knowledge and included knowledge regarding mental resilience, teaching and how to cope with stress or negative events. Finally, supervision of the peer educators was carried out twice per semester to assess progress and collect evidence of activities including activity records, adolescent posters and activity photos. The average number of activities per class was 20, ranging from 8 to 50. Each class consisted of 5.5 posters on average, with minimum values of 4 posters and maximum values of 8 posters per class.

Physiological knowledge, psychological health education, and health lifestyles were the three areas targeted. Physiological health education included growth spurts, the development of secondary sexual characteristics, acne treatment, “how to treat breast development, menstruation and dysmenorrhea for girls”, “how to deal with beard growth and spermatorrhea for boys” and cleaning of the private parts. Psychological health education involved characteristics of adolescent psychological development and “how to deal with common psychological problems such tension, anxiety and conflicts with parents, teachers and companions”; health lifestyle included a balanced diet, reasonable exercise, and keeping good sleeping patterns (see online resource in their Supplementary Materials - 10.1007/s11136-019-02309-3).

## Discussion

### Limitations and Strengths

Readers might have been surprised that no attempt was made to assess the quality of the reviews mentioned. There were a number of reasons for that. Firstly, the quality of studies was in general higher from developed countries than developing countries (although with some notable exceptions), so any assessment would have been somewhat unfair in that respect. Secondly, the quality of studies from both developed and developing countries tended to increase as time went by, so later studies were more likely to be of good quality. Thirdly, the definition of “good quality” is itself a contentious term. For some, “good quality” means Randomized Controlled Trials (RCTs). Others note that RCTs often focus on a very small number of studies, ignoring most of the literature in the area. Yet others would argue that quantitative positivistic methods can never convey the richness of what is being researched. On the other hand, purely qualitative researchers have difficulty making claims about outcomes, as they only have subjective perceptions on which to rely. However, it is clear that only one of the reviews mentioned here (in physical activity) specifically cited effect sizes, while others gave probabilities or mean differences, but not standardized mean differences. For the future, one would hope and expect that all studies would give effect sizes for each relevant variable studied.

One might naively assume that as reviews (especially systematic reviews and meta-analyses) follow a standard pattern, they are comparable. However, inspection of the above shows very quickly that even these are very variable– in terms of the focus, the keywords chosen for the search, the time range of the search, the number and type of databases searched and the inclusion criteria for studies. Given this, it is perhaps surprising that there is as much agreement between them as is actually seen.

Regarding the reviews and studies themselves, in relatively few cases was longer-term follow-up part of the project. There is mention of follow-up over periods as long as 18 months [11, 14, 18], but this was rare. Thus, we do generally not know if there were any enduring effects. Of course, it might not be reasonable to expect enduring effects, especially if the intervention was stopped, since many other life events would intervene and cloud the picture. Nonetheless, some degree of sustainability of outcomes, both with continuing intervention and without it, would be welcome. Those projects which have continuity arrangements, with clients in receipt of service being recruited to be new peer helpers, clearly have advantages here, not least since there is evidence of benefit to peer helpers as well as the helped.

The strength here is the focus here not just on outcomes, but also on implementation– i.e., *how* that outcome was achieved and in what context. Knowledge of “what works” is all very well, but it is only the first step towards delivering services that make a difference. For this latter, detailed awareness of how to implement in any specific context is needed. Project leaders need to be sharply aware of the importance of constant re-adjustment to meet developing challenges during the course of the project.

### Relationship to Other Areas

Overall, despite some reviews finding limited gains, there was strong evidence of the effectiveness of peer intervention in obesity and physical activity. This places peer intervention in obesity and physical activity alongside other effective areas: Sexual Health, HIV/AIDS, Cancer, Diabetes, Chronic Health Conditions, Heart Conditions, Stroke, Drug Use, Smoking, Breastfeeding and Nutrition. In Obesity, 36% of studies were from developing countries (this relatively high proportion reflecting the global concern with this issue). In Physical Activity, only 13% of studies were from developing countries (this relatively low proportion reflecting a lesser preoccupation with preventive studies in developing countries) [2].

### Implementation

Eighteen variables in ensuring effective peer interventions were identified: Cultural Context, Recruiting Organizations, Pilot Consultations, Peer Helper Role, Mode of Delivery, Peer Helper Recruitment, Peer Helper Training, Assessment of Training Effects, Equipment including Digital Technology, Matching and First Contact, Frequency and Duration, Activity, Contract, Record-keeping and Monitoring, Support for Peer Helpers, Implementation Fidelity, Acknowledgement and Remuneration, Retention and Longer Term Planning. Further details will be found in [2].

### Evaluation

Practitioners and researchers are encouraged to evaluate projects using a range of types of measure - preferably at least one from each of the following five categories: Descriptives; Subjective Perceptions; Knowledge, Attitudes and Behavioral Intentions; Behaviors; Physiological Indicators. Combinations of different types of outcome measure are likely to lead to more robust evaluations. Further details will be found in [2].

## Conclusion

This paper found that peer intervention in obesity and physical activity showed quite strong evidence of effectiveness. Had all studies followed the implementation and evaluation practice recommendations outlined above, it may be that the strength of evidence would have improved. However, following all those recommendations would have increased cost (even assuming it were possible), which raises the issue of cost effectiveness. Peer intervention may be cheaper than professional intervention, but it is not free, not least since professional time might be taken up with organizing, operating and monitoring the intervention. Additionally, peer intervention may occur more frequently than professional intervention, albeit at lower unit cost. Very few studies addressed the issue of cost-effectiveness, but in such varied contexts that they were difficult to summarize. There is no doubt that cost-effectiveness and longer-term follow-up are the two questions urgently needing more research, together with clear citing of effect sizes in study reports. In addition, physical activity research may need more attention in developing countries.

## Key References

- Tweed LM., Rogers EN., Kinnafick FE. Literature on peer-based community physical activity programmes for mental health service users: A scoping review. *Health Psychol Rev*. 2020; 15(2), 287–313. 10.1080/17437199.2020.1715812.
-
  - A scoping review focused on mental health service users. About half the studies reported perceived mental health benefits (others did not necessarily look for these). No physiological indicators. Nothing about implementation.
- Huitink M, Poelman MP, Seidell JC, Dijkstra SC. The healthy supermarket coach: Effects of a nutrition peer-education intervention in Dutch supermarkets involving adolescents with a lower education level. *Health Educ Behav.* 2021; 48(2) 150–159. 10.1177/1090198120957953.
-
  - A powerful study locating coaching actually at the point of temptation, with considerable detail about implementation. Focused only on 12–14-year-olds. However, gains compared to controls were mostly based on self-report.
- Lim S, Lee WK, Tan A, Chen ML, Tay CT, Sood S, et al. Peer-supported lifestyle interventions on body weight, energy intake, and physical activity in adults: A systematic review and meta-analysis. *Obes Rev.* 2021; 22, e13328. 10.1111/obr.13328.
-
  - A relatively recent traditional systematic analysis of peer interventions on body weight, energy intake and physical activity in adults, searching many databases and including a substantial number of articles. Positive effects including on physiological indices, but nothing about implementation.
- McHale F, Ng K, Taylor S, Bengoechea E, Norton C, O’Shea D, et al. A systematic literature review of peer-led strategies for promoting physical activity levels of adolescents. *Health Educ Behav.* 2022; *49*(1), 41–53. 10.1177/10901981211044988.
-
  - Focused only on adolescents, half the studies reported gains in the school setting, but were largely dependent on self-report. Gender-specific interventions showed great promise. Nothing about implementation.
- Nguyen NM, Dibley MJ, Tang HK, Alam A. Effectiveness of peer-led programs for overweight and obesity in children: Systematic review and meta-analysis. *Int J Obes.* 2022; 46(12), 2070–2087. 10.1038/s41366-022-01219-8.
-
  - A relatively recent meta-analysis focusing on obesity in children, but only from high-income countries and with short minimum program duration. Outcome measure was BMI. Longer interventions produced better effects. Nothing about implementation.

## Electronic Supplementary Material

Below is the link to the electronic supplementary material.

Supplementary Material 1

## Data Availability

No datasets were generated or analysed during the current study.
