# Supplementary material for: Peer Intervention in Obesity and Physical Activity: Effectiveness and Implementation
Source: Curr Obes Rep. 2025 Apr 3;14(1):29. doi: 10.1007/s13679-025-00625-z (PMC11965182; doi:10.1007/s13679-025-00625-z)
Supplement: Supplementary file 1 — Supplementary Material 1 [file 13679_2025_625_MOESM1_ESM.docx]

**Peer Intervention in Obesity and Physical Activity: Effectiveness and Implementation**

Keith J Topping

University of Dundee

k.j.topping@dundee.ac.uk

Current Obesity Reports

**Appendix 1: References to Single Studies Not Otherwise Mentioned (Online Only)**

***Obesity***

Allicock, M., Haynes-Maslow, L., Carr, C., Orr, M., Kahwati, L. C., Weiner, B. J., et al. (2013). Training veterans to provide peer support in a weight-management program: MOVE! *Preventing Chronic Disease, 10*, 130084. http://dx.doi.org/10.5888/pcd10.130084

Ariga, R. A., Astuti, S. B., Ariga, F. A., & Ariga, S. (2020). Improved knowledge and attitude about healthy snack at school through peer education. *International Journal on Advanced Science Engineering Information Technology, 10*(4).

Bell, S. L., Audrey, S., Cooper, A. R., Noble, S., & Campbell, R. (2017). Lessons from a peer-led obesity prevention programme in English schools. *Health Promotion International, 32*, 250–259. doi: 10.1093/heapro/dau008

Cherrington, A. L., Willig, A. L., Agne, A. A., Fowler, M. C., Dutton, G. R., & Scarinci, I. C. (2017). Development of a theory-based, peer support intervention to promote weight loss among Latina immigrants. *BMC Obesity, 2*, 17. doi: 10.1186/s40608-015-0047-3

Cui, Z., Shah, S., Yan, L., Pan, Y. P., Gao, A. Y., Shi, X. Y., et al. (2012). Effect of a school-based peer education intervention on physical activity and sedentary behaviour in Chinese adolescents: A pilot study. *BMJ Open, 2*, e000721. doi:10.1136/bmjopen-2011-000721

Damghanian, A., Sharietpanahi, G., Khieltash, A., & Barahimi, H. (2019). Effect of peer education on physical activity and nutrition among Iranian adolescents. *Asian Journal of Social Health and Behavior, 2*, 52-57. doi: 10.4103/SHB.SHB_8_19

Darise, D. S., Hidayanty, H., Suriah, Hadju, V., Bahar, B., & Ibrahim, E. (2021). The effect of peer education on the behavior of snack consumption in Gorontalo Regency. *Journal La Edusci, 2*(1), 6-13). doi: 10.37899/journallaedusci.v2i1.291

de Souza, R., Dauner, K. N., Goei, R., LaCaille, L., Kotowski, M. R., Schultz, J. F., et al. (2013). An evaluation of the peer helper component of *Go*!: A multimessage, multi-“step” obesity prevention intervention. *American Journal of Health Education, 45*(1), 12-19. https://doi.org/10.1080/19325037.2013.853001

Eickman, L., Betts, J., Pollack, L., Bozsik, F., Beauchamp, M., & Lundgren, J. (2018). Randomized controlled trial of REbeL: A peer education program to promote positive body image, healthy eating behavior, and empowerment in teens. *Eating Disorders*, *26*(2), 127–142. https://doi.org/10.1080/10640266.2017.1349005

Fiks, A. G., Gruver, R. S., Bishop-Gilyard, C. T., Shults, J., Virudachalam, S., Suh, A. W., et al. (2017). A social media peer group for mothers to prevent obesity from infancy: The Grow2Gether randomized trial. *Childhood Obesity, 13*(5), doi: 10.1089/chi.2017.0042

Foley, B. C., Shrewsbury, V. A., Hardy, L. L., Flood, V. M., Byth, K., & Shah, S. (2017). Evaluation of a peer education program on student leaders’ energy balance-related

behaviors. *BMC Public Health, 17*, 695. doi: 10.1186/s12889-017-4707-8

Goldfinger, J. Z., Arniella, G., Wylie-Rosett, J., & Horowitz, C. R. (2008). Project HEAL: Peer education leads to weight loss in Harlem. *Journal of Health Care for the Poor and Underserved, 19*(1), 180–192. doi: 10.1353/hpu.2008.0016

Gruver, R. S., Bishop-Gilyard, C. T., Lieberman, A., Gerdes, M., Virudachalam, S., Suh, A. W., et al. (2016). A social media peer group intervention for mothers to prevent obesity and promote healthy growth from infancy: Development and pilot trial. *JMIR Research Protocols, 5*(3), e159. doi: 10.2196/resprot.5276

Hadi, A. J., Hadju, V., Suriah, Indriasari, R., Sudargo, T., Nyorong, M., et al. (2019). Model of peer intervention assessment of nutritional educator in the efforts to change behaviour in decreasing overweight in integrated Islamic elementary schools at Makassar. *Indian Journal of Public Health Research & Development, 10*(9), 613-618.

Henström, M., Duncanson, K., Collins, C. E., Ashton, L. M., Davidson, E., & Ball, R., (2022). Online reach and engagement of a child nutrition peer‑education program (PICNIC):

Insights from social media and web analytics. *BMC Public Health, 22,* 836. https://doi.org/10.1186/s12889-022-13252-3

Hibbert, C., Trottier, E., Boville, M., Hahn, M., Hernandez, A., Siddiqui, A. (2021). The effect of peer support on knowledge and self‑efficacy in weight management: A prospective clinical trial in a mental health setting. *Community Mental Health Journal, 57*, 979–984. https://doi.org/10.1007/s10597-020-00703-7

Imanaka, M., Ando, M., Kitamura, T., & Kawamura, T. (2013). Effectiveness of web-based self-disclosure peer-to-peer support for weight loss: Randomized controlled trial. *Journal of Medical Internet Research, 15*(7), e136. http://www.jmir.org/2013/7/e136

Kim, H. W., Ray, C. D., & Veluscek, A. M. (2017). Complementary support from facilitators and peers for promoting mhealth engagement and weight loss. *Journal of Health Communication*. doi: 10.1080/10810730.2017.1373876

Kiss, Z. S., Vitrai, J., Tak´acs, J., Luk´acs, J´. A., Falus, A., & Feith, H. J. (2024). Peer education program to improve fluid consumption in primary schools: Lessons learned from an innovative pilot study. *Heliyon, 10*, e26769. https://doi.org/10.1016/j.heliyon.2024.e26769

Leahey, T. M., Huedo-Medina, T. B., Grenga, A., Gay, L., Fernandes, D., Denmat, Z., et al. (2020). Patient-provided e-support in reduced intensity obesity treatment: The INSPIRE randomized controlled trial. *Health Psychology*, *39*(12), 1037. https://doi.org/10.1037/hea0000996

Ma'at, I., Harris, K. A., Harris, G. A., & Rodney, P. (2015). Y.E.S. 4 Health: A peer education approach to prevention of diabetes in African American adolescents. *DePaul Journal of Health Care*. https://via.library.depaul.edu/jhcl/vol10/iss1/10

Manggabarani, S., Said, I., Hadi, A. J., Saragih, R., Cristandy, M., & Januariana, N. E. (2020). The effectivity of peer education module on knowledge, attitude, and fast food consumption in adolescents. *Journal of Health Promotion and Behavior, 5*(1), 35-43. https://doi.org/10.26911/thejhpb.2020.05.01.05

## Masri, E., Tunnisa, M. A., & Ilham, D. (2023). The effectiveness of peer education and Instagram nutrition education on changes in knowledge and consumption of risky foods in students. *PROMOTOR: Jurnal Mahasiswa Kesehatan Masyarakat, 6(*6), 626-636. https://doi.org/10.32832/pro.v6i6.464

Muzaffar, H., Nikolaus, C. J., & Sharon M. Nickols-Richardson, S. (2020). Students’ reflections on an experiential learning rotation with the peer-education About Weight Steadiness Club program. *Pedagogy in Health Promotion,* [*9*(2](https://journals.sagepub.com/toc/phpa/9/2)).

<https://doi.org/10.1177/2373379920960647>

[Nikolaus, C. J](https://www.webofscience.com/wos/author/record/41033840)., [Liguori, C. A](https://www.webofscience.com/wos/author/record/25297664)., [Winslow, A. N](https://www.webofscience.com/wos/author/record/5563800).,  & [Nickols-Richardson, S. M](https://www.webofscience.com/wos/author/record/12500987). (2016). Peer-education About Weight Steadiness (PAWS club): Pilot test of family menu planning lesson for parents and their young adolescents. *FASEB Journal, 30*(Supplement 1).

https://doi.org/10.1096/fasebj.30.1_supplement.896.18

Quintiliani, L. M., & Whiteley, J. A. (2016). Results of a nutrition and physical activity peer counseling intervention among nontraditional college students. *Journal of Cancer Education, 31*(2), 366–374. doi:10.1007/s13187-015-0858-4

Saez, L., Legrand, K., Alleyrat, C., Ramisasoa, S., Langlois, J., Muller L., et al. (2018). Using facilitator-receiver peer dyads matched according to socioeconomic status to promote behaviour change in overweight adolescents: A feasibility study. *BMJ Open, 8*(6), e019731. doi: 10.1136/bmjopen-2017-019731

Slawson, D. L., Dalton, W. T., Dula, T. M., Southerland, J., Wang, L., Littleton, M. A., et al. (2015). College students as facilitators in reducing adolescent obesity disparity in Southern Appalachia: Team Up for Healthy Living. *Contemporary Clinical Trials, 43*, 39-52. doi: 10.1016/j.cct.2015.04.012

Stock, S., Miranda, C., Evans, S., Plessis, S., Ridley, J., Yeh, S., et al. (2007). Healthy Buddies: A novel, peer-led health promotion program for the prevention of obesity and eating disorders in children in elementary school. *Pediatrics, 120*(4), e1059–e1068. https://doi.org/10.1542/peds.2006-3003

Vairano, M. P., Krauss, P. R., D’Agnese, P., Iavarone, F., Rice, R., Strangio, F., et al. (2021). The prevention of obesity starting from the kindergarten by means of peer educator mothers and *WhatsApp* messages. *Annali di Igiene: Medicina Preventiva e di Comunità*, *34*(2). doi: 10.7416/ai.2021.2453

***Physical Activity***

Boudreau, F., Moreau, M., & Côté, J. (2016). Effectiveness of computer tailoring versus peer support web-based interventions in promoting physical activity among insufficiently active Canadian adults with type 2 diabetes: Protocol for a randomized controlled trial. *JMIR Research Protocols, 5(1), e20.* doi: 10.2196/resprot.5019

Boyle, J., Mattern, C. O., Lassiter, J. W., & Ritzler, J. A. (2011). Peer 2 Peer: Efficacy of a course-based peer education intervention to increase physical activity among college students. *Journal of American College Health, 59*(6), 519-529. https://doi.org/10.1080/07448481.2010.523854

Brown, K. N., Wengreen, H. J., Beals, K. A., & Heath, E. M. (2024). Effects of peer-education on knowledge of the female athlete triad among high school track and field athletes: A pilot study. *Women in Sport and Physical Activity Journal, 24*(1), 1-6. https://doi.org/10.1123/wspaj.2014-0058

Cai, X., Qiu, S. H., Luo, D., Li, R. X., Liu, C. Y., Lu, Y. H., et al. (2022). Effects of peer support and mobile application‑based walking programme on physical activity and physical function in rural older adults: A cluster randomized controlled trial. *European Geriatric Medicine, 13*, 1187–1195. https://doi.org/10.1007/s41999-022-00682-w

Castro, C. M., Pruitt, L. A., Buman, M. P., & King, A. C. (2011). Physical activity program delivery by professionals versus volunteers: the TEAM randomized trial. *Health Psychology, 30*(3), 285–294. doi: 10.1037/a0021980

Conley, C., Randolph, S., Hardison-Moody, A., Gonzalez-Guarda, R., Fisher, E. B., & Lipkus, I. (2023). Feasibility of dyadic peer support to augment a church-based healthy lifestyle programme. *Health Education Journal*, 82(7), 725-738. <https://doi.org/10.1177/00178969231185652>

Cox, K. L., Cyarto, E. V., Etherton-Beer, C., Ellis, K. A., Alfonso, H., Clare, L., et al. (2017). A randomized controlled trial of physical activity with individual goal-setting and volunteer mentors to overcome sedentary lifestyle in older adults at risk of cognitive decline: The INDIGO trial protocol. *BMC Geriatrics, 17*, 215. doi: 10.1186/s12877-017-0617-y

Crozier, A., Porcellato, L., Buckley, B. J. R., & Watson, P. M. (2020). Facilitators and challenges in delivering a peer-support physical activity intervention for older adults: A qualitative study with multiple stakeholders. *BMC Public Health, 20*, 1904. https://doi.org/10.1186/s12889-020-09990-x.

d’Arripe-Longueville, F., Gernigon, C., Huet, M. L., Winnykamen, F., & Cadopi, M. (2002). Peer-assisted learning in the physical activity domain: Dyad type and gender differences. *Journal of Sport and Exercise Psychology*, *24*(3), 219-238. doi:  https://doi.org/10.1123/jsep.24.3.219

Esentürk, O. K., & Güngör, N. B. (2020). The effect of peer-mediated adaptive physical activity program on problem behaviors of mentally handicapped students. *Journal of Education and Learning, 9*(3). doi: 10.5539/jel.v9n3p163

Fallace, P., Aiese, P., Bianco, E., Bolognini, I., Costa, M. P., Esposito, R., et al. (2019). Peer education strategies for promoting prevention of doping in different populations. *Annali di Igiene: Medicina Preventiva e di Comunita, 31*, 556-575. doi: 10.7416/ai.2019.2316

Gobbi, E., Greguol, M., & Carraro, A. (2018). Brief report: Exploring the benefits of a peer-tutored physical education programme among high school students with intellectual disability. *Journal of Applied Research in Intellectual Disabilities*, *31*(5), 937-941.

https://doi.org/10.1111/jar.12437

Graham, C. R., Larstone, R., Griffiths, B., de Leeuw, S., Anderson, L., Powell-Hellyer, S., et al. (2007). Development and evaluation of innovative peer-led physical activity programs for mental health service users. *Nervous and Mental Disease, 205*, 840–847. doi: 10.1097/NMD.0000000000000746

Guest, D. D., Thorpe, M. P., Mojtahedi, M. C., Richey, A., Mailey, E., Kedem, L. E., et al. (2010). Peer education initially helps prevent weight changes in freshman women. *The FASEB Journal*, *24*(S1), 564-510. https://doi.org/10.1096/fasebj.24.1_supplement.564.10

Haghparast, A., Rohani, C., Vasli, P., Salmani, F., & Marzaleh, M. A. (2020). Effect of two educational methods of lecturing and peer group on physical activity among 12-15-year-old students in health promoting schools. *Iran Red Crescent Medical Journal, 22*(9), e59. doi: 10.32592/ircmj.2020.22.9.59

Haidar, A., Ranjit, N., Archer, N., & Hoelscher, D. M. (2019). Parental and peer social

support is associated with healthier physical activity behaviors in adolescents: A cross-sectional analysis of Texas School Physical Activity and Nutrition (TX SPAN) data. *BMC Public Health, 19*(1), 640. doi: 10.1186/s12889-019-7001-0

Henström, M., Duncanson, K., Collins, C. E., Ashton, L. M., Davidson, E., & Ball, R. (2022).

Online reach and engagement of a child nutrition peer-education program (PICNIC): Insights from social media and web analytics. *BMC Public Health, 22*(1), 836. doi: 10.1186/s12889-022-13252-3

Henwood, T., Pinchbeck, J., & Leicht, A. (2011). Using peer educator delivered seminars to improve the level of physical activity among older adults: A pilot investigation. *Journal of Rural and Tropical Public Health, 10*, 1-7. http://www.jcu.edu.au/jrtph/vol/JRTPH_Vol10_p1-7_Henwood.pdf

Hyndman, B. P., & Telford, A. (2015). Should educators be 'wrapping school playgrounds in cotton wool' to encourage physical activity? Exploring primary and secondary students' voices from the school playground. *Australian Journal of Teacher Education (Online)*, *40*(6), 60-84. https://search.informit.org/doi/abs/10.3316/ielapa.276778285485034

King, A, C., Campero, M. I., Sheats, J. L., Sweet, C. M. C., Hauser, M. E., Garcia, D., et al. (2020). Effects of counseling by peer human advisors vs computers to increase walking in underserved populations: The COMPASS randomized clinical trial. *JAMA Internal Medicine, 180*(11), 1481-1490. doi: 10.1001/jamainternmed.2020.4143

Klavina, A., & Block, M. E. (2008). The effect of peer tutoring on interaction behaviors in inclusive physical education. *Adapted Physical Activity Quarterly*, *25*(2), 132-158. https://doi.org/10.1123/apaq.25.2.132

Kondo, M., & Kato, H. (2023). A longitudinal study of the impact of the peer support programme on a Japanese male-dominated high school through 6 years practices. *European Journal of Psychology and Educational Research*, *6*(2), 85-96. https://doi.org/10.12973/ejper.6.2.85

Kondo, M., & Kato, H. (2024). The impact of the peer support programme on interpersonal relationship, self-esteem, general health questionnaire and adaptation scale for school environments on six spheres among Japanese high school pupils. *European Journal of Psychology and Educational Research*, *7*(1), 1-10. https://doi.org/10.12973/ejper.7.1.1

Kulik, N., Ennett, S. T., Ward, D. S., Bowling, J. M., Fisher, E. B., & Tate, D. F. (2015). Brief report: A randomized controlled trial examining peer support and behavioral weight loss treatment. [*Journal of Adolescence*](https://www.sciencedirect.com/journal/journal-of-adolescence)*,* [*44*](https://www.sciencedirect.com/journal/journal-of-adolescence/vol/44/suppl/C), 117-123. https://doi.org/10.1016/j.adolescence.2015.07.010

Lawler, M., Heary, C., & Nixon, E. (2020). Peer support and role modelling predict physical activity change among adolescents over twelve months. *Journal of Youth and Adolescence, 49*, 1503–1516. https://doi.org/10.1007/s10964-019-01187-9

Logsdon, P., Samudre, M., & Kleinert, H. (2018). *A qualitative study of the impact of peer networks and peer support arrangements in project pilot schools*. Lexington KY: Human Development Institute, University of Kentucky.

Mathews, E., Sauzet, O., & Thankappan, K. R. (2021). Effectiveness of a physical activity intervention program using peer support among sedentary women in Thiruvananthapuram City, India: Results of a non-randomized quasi experimental study. *Wellcome Open Research, 6*, 87. https://doi.org/10.12688/wellcomeopenres.16618.1

Middleton, R., Metusela, C., Marriott-Statham, K., Ferguson, C., & Davidson, P. M. (2023).

The engagement of older people living with chronic lung disease in a peer

support community-based exercise programme: A qualitative study. *Health Expectations: An International Journal of Public Participation in Health Care and Health Policy, 26*(6), 2409-2417. doi: 10.1111/hex.13847

Oli, N., Vaidya, A., Eiben, G., & Krettek, A. (2019). Effectiveness of health promotion regarding diet and physical activity among Nepalese mothers and their young children: The Heart-health Associated Research, Dissemination, and Intervention in the Community (HARDIC) trial. *Global Health Action, 12*(1), 1670033, doi:

10.1080/16549716.2019.1670033

Olsen, K., Martin Ginis, K. A., Lawrason, S., McBride, C. B., Walden, K., Le Cornu, L. C., et al. (2023). Assessing the reach, effectiveness, adoption, implementation, and maintenance of the ProACTIVE SCI physical activity counselling intervention among physiotherapists and SCI peer coaches during the transition from rehabilitation to community. *Frontiers in Neurology, 14*, 1286129. doi: 10.3389/fneur.2023.1286129

Owen, M. B., Kerner, C., Taylor, S. L., Noonan, R. J., Newson, L., Kosteli, M., et al. (2018). The feasibility of a novel school peer-led mentoring model to improve the physical activity levels and sedentary time of adolescent girls: The Girls Peer Activity (G-PACT) project. *Children, 5*, 67. doi: 10.3390/children5060067

Sebire, S. J., Jago, R., Banfield, K., Edwards, M. J., Campbell, R., Kipping, R., et al. (2018). Results of a feasibility cluster randomised controlled trial of a peer-led school-based

intervention to increase the physical activity of adolescent girls (PLAN-A). *International Journal of Behavioral Nutrition and Physical Activity, 50*. https://doi.org/10.1186/s12966-018-0682-4

Shrewsbury, V. A., Venchiarutti, R. L., Hardy, L. L., Foley, B. C., Bonnefin, A., Byth, K., et al. (2020). Impact and cost of the peer-led "Students as LifeStyle Activists" programme in high schools. *Health Education Journal*, 79(1), 3-20. https://doi.org/10.1177/0017896919856050

Staiano, A. E., Abraham, A. A., & Calvert, S. L. (2013). Adolescent Exergame play for weight loss and psychosocial improvement: A controlled physical activity intervention. *Obesity, 21*, 598-601. doi: 10.1038/oby.2012.143

Stanish, H. I., & Temple, V. A. (2012). Efficacy of a peer-guided exercise programme for adolescents with intellectual disability. *Journal of Applied Research in Intellectual Disabilities*, *25*(4), 319-328. https://doi.org/10.1111/j.1468-3148.2011.00668.x

Stathi, A., Withall, J., Thompson, J. L., Davis, M. G., Gray, S., De Koning, J., et al. (2020). Feasibility trial evaluation of a peer volunteering active aging intervention: ACE (Active, Connected, Engaged). *The Gerontologist, 60*(3), 571-582. doi: 10.1093/geront/gnz003

Steward, I. P., Young, E. S., Dogra, S. A., Stamp, E., Daly-Smith, A., Siddique, K., et al. (2023). How to develop young physical activity leaders? A Delphi study. *PLoS ONE 18*(9), e0286920. https://doi.org/10.1371/journal.pone.0286920

Thi Nguyen, N. T., Tang, H. K., Nguyen, M. N., Dibley, M. J., & Alam, N. A. (2022). Effect of a peer-led education intervention on dietary behaviour and physical activity among adolescents in Ho Chi Minh City, Vietnam: A pilot study. *European Journal of Clinical Nutrition,* 76, 1590–1593. https://doi.org/10.1038/s41430-022-01140-3

van de Kop, H., den Uil, A., Nauta, J., Toussaint, H., Busch, V., Verhoeff, A., et al. (2022). Evaluation of an asset-based, participatory physical activity promotion intervention in Dutch adolescents: A parallel group randomized trial. *European Journal of Public Health, 32*, Supplement 2, P03-17, ckac095-053.

Wolfe, J. (2023). *The impact of peer interventions on physical activity for individuals living with mental illness*. Yale Medicine Thesis Digital Library. 4206. https://elischolar.library.yale.edu/ymtdl/4206

Yan, Z., Finn, K., Cardinal, B. J., & Bent, L. (2014). Promoting health behaviors using peer education: A demonstration project between International and American college students. *American Journal of Health Education*, *45*(5), 288-296. https://doi.org/10.1080/19325037.2014.932727

Yan, Z., Finn, K., & Corcoran, M. (2015). Using peer education to promote balance, fitness, and physical activity among individuals with intellectual disabilities. [*American Journal of Health Studies*](file:///D:\OFFICIAL%20BUSINESS\Publications\Book%20Peer%20Interventions%20for%20Health%20and%20Wellbeing\American%20Journal%20of%20Health%20Studies)*, 30*(4). https://doi.org/10.47779/ajhs.2015.185

Yusuf, R. A., Mont, D. F., Lin, W. H., & Chen, H. J. (2021). Adolescents' physical activity and the association with perceived social support of parents and peers in Indonesia. *Asia-Pacific Journal of Public Health, 33*(4), 388-395. doi: 10.1177/1010539521997255

Zhang, J. W., Brackbill, D., Yang, S., Becker, J., Herbert, N., & Centola, D. (2016). Support or competition? How online social networks increase physical activity: A randomized controlled trial. *Preventive Medicine Reports, 4*, 453–458. http://dx.doi.org/10.1016/j.pmedr.2016.08.008
